# Extracellular vesicle miR-425-5p promotes visceral fat reduction via DACT1 suppression in SGLT2i-treated diabetes

**DOI:** 10.3389/fendo.2025.1725625

**Published:** 2025-12-09

**Authors:** Jae-Hyung Park, Thi Nhi Nguyen, Hye Min Shim, Yun-Ui Bae, Gyeong Im Yu, Junho Kang, Eun Yeong Ha, Hochan Cho

**Affiliations:** 1 Department of Physiology, Keimyung University School of Medicine, Daegu, Republic of Korea; 2 Department of Research, Keimyung University Dongsan Medical Center, Daegu, Republic of Korea; 3 Department of Internal Medicine, Keimyung University School of Medicine, Daegu, Republic of Korea

**Keywords:** extracellular vesicle, microRNA, sodium-glucose transporter 2 inhibitor, type 2diabetes, obesity, adipocyte, miR-425-5p, dact1

## Abstract

**Objective:**

In type 2 diabetes (T2D), plasma-derived extracellular vesicle (EV)-microRNAs (miRNAs) contribute to insulin resistance and β-cell dysfunction. Sodium-glucose cotransporter 2 inhibitors (SGLT2i) lower blood glucose by promoting urinary glucose excretion and are associated with weight loss, although the underlying mechanisms remain unclear.

**Methods:**

This study was conducted in two phases. In the screening phase, plasma-derived EV miRNA profiles were analyzed by small RNA sequencing in 13 patients with newly diagnosed, treatment-naive T2D at baseline and after 12 months of SGLT2i therapy. In the validation phase, expression of selected miRNAs was quantified by real-time PCR in an independent cohort of 30 patients at baseline and after 6 and 12 months of treatment. The functional roles of candidate miRNAs were examined in 3T3-L1 adipocytes.

**Results:**

Small RNA sequencing identified 18 plasma-derived EV miRNAs exhibiting >1.5-fold expression changes after 12 months of SGLT2i therapy. Quantitative PCR confirmed that five EV miRNAs were significantly upregulated post-treatment. Among these, miR-425-5p showed a strong inverse correlation with waist circumference and visceral fat area. Functional assays in 3T3-L1 adipocytes demonstrated that miR-425-5p suppressed adipogenic differentiation and lipid accumulation by downregulating DACT1, one of its predicted target genes, and reducing DACT1-reporter activity.

**Conclusion:**

Plasma-derived EV miR-425-5p, increased by long-term SGLT2i therapy in T2D patients, may inhibit adipocyte differentiation and lipogenesis via DACT1 suppression. These findings suggest a possible mechanistic link through which SGLT2i treatment may ameliorate visceral obesity.

## 1 Introduction

Small extracellular vesicles (EVs) include microvesicles measuring 100–1000 nm in size and exosomes measuring 30–100 nm in size. They are secreted by various cell types (1). EVs play a crucial role in intercellular communication (2). They carry molecular cargo such as proteins, lipids, and nucleic acids (e.g., microRNA (miRNA)), and deliver them to target cells, influencing various physiological and pathological processes (3). miRNAs in EVs (EV miRNA) are particularly important as they regulate gene expression in recipient cells by binding to mRNAs and then either degrading them or inhibiting their translation (4). This regulatory function of miRNAs contributes to key processes such as immune modulation, tissue repair, and cancer progression. Additionally, plasma-derived EV miRNAs have been explored as potential biomarkers for diseases such as cancer and neurodegenerative disorders due to their stability in circulation and their involvement in disease pathogenesis (5).

EV miRNAs are known to contribute to diabetes development and progression by facilitating intercellular communication that impacts insulin responsiveness, pancreatic β-cell performance, and inflammatory pathways (6). In type 2 diabetes (T2D), insulin-resistant cells like adipocytes and myocytes release EVs containing abnormally expressed miRNAs, which in turn may disrupt insulin signaling mechanisms in recipient cells (7). For example, specific miRNAs have been shown to suppress genes involved in glucose uptake and insulin sensitivity, thus contributing to metabolic dysfunction (8, 9). Additionally, EV miRNAs derived from immune cells or damaged pancreatic β-cells can promote inflammatory responses, further exacerbating insulin resistance and β-cell destruction (10, 11). This communication pathway not only accelerates diabetes progression but also links various organs to the disease’s systemic effects. Moreover, changes in expression profiles of EV miRNAs are being investigated as potential biomarkers to predict diabetes risk or monitor disease progression and treatment response (12).

Sodium-glucose cotransporter 2 inhibitor (SGLT2i) is a relatively recent class of medication approved by the FDA for treating T2D (13). Unlike traditional medication such as metformin and insulin used for treating diabetes that focuses on enhancing insulin secretion or sensitivity, SGLT2i targets the kidneys. Specifically, it inhibits sodium-glucose transporter 2 in renal tubules, reducing reabsorption of glucose into the bloodstream and promoting its excretion through urine. This insulin-independent mechanism makes SGLT2i unique, as it can lower blood glucose levels without directly impacting insulin pathways (14). In addition to improving glycemic control, SGLT2i offers several other benefits, including weight loss and blood pressure reduction, which are particularly advantageous for patients with obesity or cardiovascular risks (15, 16). Moreover, it has been shown that SGLT2i can provide renal and cardiovascular protective effects, making it a valuable treatment option for managing both diabetes and its complications (17, 18). SGLT2i has recently been reported to be effective for weight loss, although its mechanisms and main targets are not well known (19, 20). In addition, the effect of SGLT2i on expression profile of EV miRNAs (21, 22), which have been recognized as important in diabetes, is not clearly known.

This study examined how long-term SGLT2i therapy affects body weight and EV–associated miRNA expression in overweight patients with T2D. Among the altered EV miRNAs, we focused on miR-425-5p, which showed a strong association with visceral fat reduction. Furthermore, we explored its putative downstream target, disheveled-binding antagonist of beta-catenin 1 (DACT1), to investigate how miR-425-5p may regulate adipocyte differentiation and lipid metabolism *in vitro*.

## 2 Materials and methods

### 2.1 Participant recruitment

For small RNA sequencing–based miRNA screening, thirteen patients newly diagnosed with T2D and not previously treated with any antidiabetic medication were recruited between March 2020 and March 2022 from Keimyung University Dongsan Hospital (Daegu, Republic of Korea). Blood sampling and clinical evaluation were performed at diagnosis (baseline). Following this, patients received combination therapy with metformin and an SGLT2i. Among these, ten patients received empagliflozin (10 mg/day), and three patients received dapagliflozin (10 mg/day). All participants were re-evaluated after 12 months of treatment.

An additional cohort of thirty participants was recruited to validate selected miRNAs using quantitative PCR. Thirty patients newly diagnosed with T2D and not previously treated with any antidiabetic medication were recruited between March 2021 and March 2023 from Keimyung University Dongsan Hospital. Blood sampling and clinical evaluation were performed at diagnosis (baseline). Following this, patients received combination therapy with metformin and an SGLT2i, and were re-evaluated at 6 and 12 months of treatment. In this validation cohort, twenty-two patients received empagliflozin (10 mg/day) and eight patients received dapagliflozin (10 mg/day).

The study protocol received approval from the Institutional Review Board (IRB) of Keimyung University Dongsan Hospital (IRB No. 2018-11-001, approved on November 30, 2018). T2D was diagnosed based on the guidelines provided by the American Diabetes Association (23). Peripheral blood was collected from patients into EDTA-containing tubes (Becton Drive, Franklin Lakes, NJ, USA). All assessments were conducted after an 8-hour fast. Trained nurses measured blood pressure and anthropometric indices following standardized procedures (24). At each time point (baseline, 6 months, and 12 months), anthropometric and biochemical parameters including body weight, body mass index (BMI), waist circumference, fasting plasma glucose, glycated hemoglobin (HbA1c), fasting insulin, homeostatic model assessment of insulin resistance (HOMA-IR), lipid profile, and blood pressure were measured using standardized procedures. These clinical indices were used to evaluate metabolic changes during SGLT2i treatment.

### 2.2 Isolation and characterization of plasma-derived extracellular vesicles

EVs were extracted from patient plasma samples using the ExoQuick reagent (System Biosciences, Palo Alto, CA, USA), following the instructions provided by the manufacturer. To confirm the presence of EVs, CD63 expression was quantified using the ExoELISA-ULTRA CD63 kit (System Biosciences), as per the manufacturer’s instructions. The physicochemical characteristics of EVs were analyzed by Dynamic Light Scattering (DLS) using a Nano-ZS Zetasizer (Malvern Panalytical, UK) to determine the mean and median hydrodynamic diameters and polydispersity index (PDI). Each sample was measured in triplicate, and data was processed with Zetasizer software (version 7.11). Imaging was performed using a transmission electron microscope (H-7000B, Hitachi, Tokyo, Japan).

### 2.3 Western blot analysis

For cellular protein analysis, equal amounts of total protein were separated by SDS-PAGE and transferred to PVDF membranes. Primary antibodies against DACT1 (Thermo Fisher Scientific), PPARγ and SREBP1 (Abcam), and CEBPα and ACC1 (Santa Cruz Biotechnology) were used according to the manufacturers’ instructions.

For EV characterization, plasma-derived EV proteins were quantified using a BCA assay, and 10 μg of EV protein per lane was loaded. EV-depleted plasma, plasma EV samples, and 3T3-L1 cell lysates were analyzed. Membranes were incubated with primary antibodies recognizing EV-enriched markers (CD9, TSG101; Abcam), the lipoprotein contamination marker APOA1 (Abcam), and the cellular contamination marker GM130 (Abcam). Detection of CD9 and TSG101 and absence of GM130 confirmed EV enrichment, whereas APOA1 was detected only in EV-depleted plasma, indicating minimal lipoprotein contamination in isolated EVs. The mean EV protein yield was 6.8 ± 1.2 μg/mL plasma.

### 2.4 EV RNA-based small RNA library preparation and sequencing

EV RNAs isolated from blood samples were used to construct small RNA libraries with the SMARTer smRNA-Seq Kit for Illumina (Takara Bio, Japan). Quantification was done using the KAPA qPCR kit, and quality was confirmed with the Agilent TapeStation. All sequencing and data processing were conducted by Macrogen (Seoul, Korea). To analyze small RNA sequencing data, miRDeep2 was used to detect both known and novel miRNAs. For annotation, uniquely aligned reads were further compared against miRBase 21 and the Rfam 9.1 database to distinguish miRNAs from other non-coding RNAs.

### 2.5 Differential miRNA expression analysis and visualization

Raw miRNA read counts were normalized using the relative log expression method implemented in DESeq2. Prior to analysis, miRNAs detected in fewer than half of the samples were excluded, and only mature miRNAs were retained. To enable log2 transformation for correlation analysis, a value of 1 was added to the normalized counts. For each miRNA, baseMean and log2 fold change values were computed to compare expression between groups. Group differences were evaluated using the Wald test with a negative binomial distribution via DESeq2. All statistical analyses and plots were generated in R version 3.3.1. We utilized the miRNet platform (https://www.mirnet.ca/) for the construction of a plasma-derived EV miRNA-target interaction network. A network of key miRNAs and their target genes was constructed using interaction data from miRTarBase, TarBase, and miRecords. Nodes with a degree ≥ 2 were retained. Functional enrichment analysis, including GO and KEGG pathways, was conducted with the clusterProfiler R package (25).

### 2.6 Quantification of miRNA by quantitative real-time PCR

Plasma-derived EV RNA (approximately 2 ng) was reverse-transcribed into cDNA using the TaqMan™ MicroRNA RT Kit (Applied Biosystems, Foster City, CA, USA) according to the manufacturer’s instructions. Quantification of EV miRNAs was performed using TaqMan™ MicroRNA Assay probes (Cat. No. 4427975; Applied Biosystems), and U6 small RNA served as the internal control for normalization. For cellular mRNA analysis, total RNA (500 ng) was extracted from 3T3-L1 cells using the RNeasy Mini Kit (Qiagen, Valencia, CA, USA) and reverse-transcribed into cDNA with the High-Capacity cDNA Reverse Transcription Kit (Applied Biosystems). Expression levels of PPARγ (Mm01184322_m1), SREBP1 (Mm00550338_m1), CEBPα (Mm00514283_s1), ACC1 (Mm01304257_m1), and DACT1 (Mm01213259_m1) were quantified using TaqMan™ Gene Expression Assays (Applied Biosystems). GAPDH (Mm99999915_g1) was used as the internal control. All reactions were performed in triplicate on a QuantStudio™ 5 Real-Time PCR System (Applied Biosystems). RNA purity and concentration were evaluated using a NanoDrop 2000 spectrophotometer (Thermo Fisher Scientific); only samples with A260/A280 ratios between 1.8 and 2.1 and A260/A230 > 1.8 were used for qPCR. Amplification efficiencies ranged from 95% to 105% with correlation coefficients (R² ≥ 0.99). Mean Ct variation among technical replicates was < 0.5 cycles, indicating high reproducibility. No-template and no-RT controls produced no detectable amplification. Relative expression levels were calculated using the 2^-^ΔΔCt method, normalized to U6 (for miRNAs) or GAPDH (for mRNAs). All data were obtained from at least three independent biological replicates and are presented as mean ± SEM.

### 2.7 Transfections of miRNAs and siRNA in 3T3-L1 cells

Adipogenesis was induced using DMEM with 10% FBS, IBMX, dexamethasone, and insulin in 3T3-L1 preadipocytes. Transfections of miRNA inhibitor or mimic of miR-425-5p in the 3T3-L1 were performed using specific mirVana miRNA inhibitor or mimic, respectively (Thermo Fisher Scientific). Knockdown of DACT1 expression in 3T3-L1 cells was performed using specific Silencer siRNAs (Thermo Fisher Scientific). 50 nM miRNA or siRNA was used for the transfection with RNAiMAX reagent (Thermo Fisher Scientific). An RNAiMAX-only (mock) group was included in every experiment to control for reagent effects. The RNAiMAX: miRNA ratio was fixed at 0.2 μL RNAiMAX per 10 pmol miRNA per well in a 96-well plate, in accordance with the manufacturer’s protocol. Non-targeting scrambled oligonucleotides without sequence homology to murine genes were used as negative controls for both mimic and inhibitor experiments. Negative control (NC) was included to analyze off-target effects. Transfection efficiency was assessed by qPCR and Western blot analyses. To evaluate the cytotoxicity of miR-425-5p transfection, 3T3-L1 preadipocytes were transfected with 0, 10, 25, 50, or 100 nM of miR-425-5p mimic or inhibitor using Lipofectamine RNAiMAX. For the time-course assay, cells were treated with 50 nM and analyzed at 0, 6, 24, 48, and 72 h post-transfection. Cell viability was determined using a CCK-8 assay (Dojindo, Japan) by measuring absorbance at 450 nm with a microplate reader. Viability was normalized to untreated control cells.

### 2.8 Luciferase reporter system to validate miRNA-target binding

To evaluate the direct interaction between *DACT1* and miR-425-5p, we used the pmirGLO Dual-Luciferase miRNA Target Expression Vector (Promega, Madison, WI, USA). A 574-bp fragment of the mouse *DACT1* 3′UTR (nucleotides 2162–2735 of NM_026772.4) containing the predicted miR-425-5p binding site (5′-AAGUGCUA-3′) was cloned downstream of the firefly luciferase gene (pmirGLO/DACT1-WT-Luc). A mutant construct (pmirGLO/DACT1-Mut-Luc) was generated by substituting three seed-region bases to disrupt miR-425-5p binding. HEK293 cells were co-transfected with 200 ng of each reporter plasmid and 50 nM miR-425-5p mimic or scramble control using Lipofectamine RNAiMAX (Thermo Fisher Scientific). Twenty-four hours after transfection, firefly and Renilla luciferase activities were measured using the Dual-Luciferase Reporter Assay System (Promega) on a luminometer, and firefly signals were normalized to Renilla for transfection efficiency. HEK293 cells were used for their high transfection efficiency and low endogenous *DACT1* expression, which minimized background interference. This system allows a reliable assessment of specific miRNA–mRNA interactions under well-controlled conditions.

### 2.9 Statistics and interpretation

For statistical analyses, SPSS software (version 29.0) was used. Clinical data were compared using paired t-tests or repeated measures ANOVA and are presented as mean ± SD. For *in vitro* studies, results are expressed as mean ± SEM, and differences between two groups were assessed using two-tailed Student’s t-tests. A value of p < 0.05 was considered statistically significant. Correlations between miRNA expression and body composition were analyzed using Spearman’s rank correlation, as normality was not satisfied.

## 3 Results

### 3.1 Screening analysis of patients with T2D treated with SGLT2i for 12 months

**Table 1** shows characteristics of patients with T2D. These patients were newly diagnosed, treatment-naive T2D patients who subsequently received metformin and SGLT2i therapy. After SGLT2i, empagliflozin or dapagliflozin, treatment for 12 months, fasting blood glucose levels and HbA1c levels, as well as weight, BMI, and waist circumference, significantly decreased. However, lipid profiles and blood pressure did not change significantly. EV miRNA expression profiling was also conducted in the screening cohort using paired plasma samples obtained before and after 12 months of SGLT2i treatment.

**Table 1:** Patient characteristics before and after SGLT2i treatment for screening experiment.

Characteristic	T2D+S0	T2D+S12	*P*-value
Number (Male/Female)	13 (7/6)	13 (7/6)	–
Age (years)	55.17 ± 9.58	56.20 ± 9.58	–
Weight (kg)	74.18 ± 10.35	72.08 ± 8.42	<0.001
BMI (kg/m^2^)	27.83 ± 2.53	25.97 ± 2.93	0.004
Waist circumference (cm)	93.12 ± 11.05	87.69 ± 10.93	0.048
HbA1c (%)	7.65 ± 0.73	6.68 ± 0.66	<0.001
Fasting glucose (mg/dL)	149.15 ± 28.31	125.24 ± 21.72	0.024
Systolic blood pressure (mmHg)	135.81 ± 19.27	129.31 ± 19.05	0.068
Diastolic blood pressure (mmHg)	83.08 ± 15.72	80.67 ± 14.25	0.483
Total cholesterol (mg/dL)	163.54 ± 33.27	150.18 ± 27.15	0.156
Triglyceride (mg/dL)	136.29 ± 31.52	142.76 ± 37.21	0.397
HDL (mg/dL)	49.81 ± 11.49	52.11 ± 10.93	0.462
LDL (mg/dL)	89.37 ± 21.14	80.46 ± 26.18	0.069

Values are expressed as number or mean ± SD. A value of p < 0.05 was considered statistically significant. BMI, body mass index; HbA1c, hemoglobin A1c; T2D+S0, type 2 diabetes before SGLT2i treatment; T2D+S12, T2D after 12 months of SGLT2i treatment.

### 3.2 Effect of 12-month SGLT2i treatment on EV miRNA expression in T2D

After extracting EVs from plasma samples, we verified characteristics of EVs (26). Transmission electron microscopy showed round, membrane-bound vesicles with an average diameter of 159.5 ± 34.5 nm (**Supplementary Figure S1A**). Dynamic light scattering (DLS) further revealed a median particle size of 179.8 ± 73.5 nm (**Supplementary Figure S1D**), consistent with the expected size range of plasma-derived EVs. The mean EV protein yield was 6.8 ± 1.2 μg/mL plasma as determined by BCA assay. Western blot analysis demonstrated clear expression of EV-enriched markers CD9 and TSG101 in EV samples, while GM130, a Golgi marker, was absent, indicating minimal cellular contamination (**Supplementary Figures S1B, C**). In contrast, ApoA1, a lipoprotein marker, was detected only in EV-depleted plasma but not in EV fractions, confirming low lipoprotein contamination after EV isolation. CD63 expression, assessed by ELISA in EV-depleted plasma and EVs, was significantly enriched in both EV samples (**Supplementary Figure S1E**). Collectively, the vesicle morphology, size distribution, positive EV marker expression (CD9, TSG101, CD63), and absence of negative markers (GM130, ApoA1 in EVs) confirm that our EV preparations meet the characterization criteria recommended by MISEV 2023. Detailed characterization parameters are summarized in **Supplementary Table S1**.

**Figure 1:**
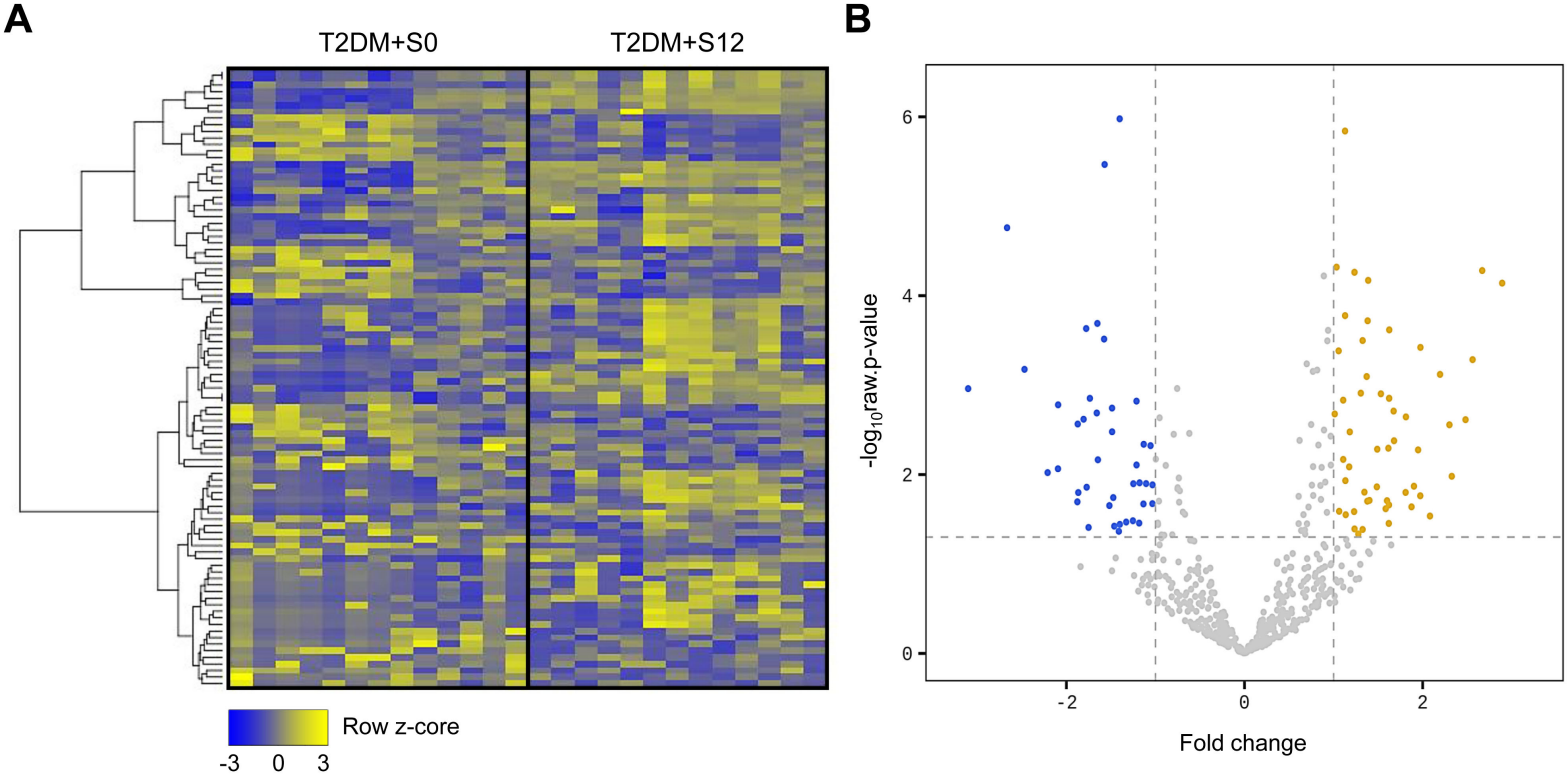
Changes in plasma-derived EV miRNA expression by SGLT2 inhibitor treatment. **(A)** Heatmap displaying z-score distributions of EV miRNAs in T2D patients (n = 13) before and after 12 months of SGLT2i treatment. Yellow and blue indicate upregulated and downregulated miRNAs, respectively. **(B)** Volcano plot illustrating 52 miRNAs with increased expression (yellow) and 39 with decreased expression (blue), each showing a fold change >1.5 after 12 months of SGLT2i treatment compared to baseline. T2D+S0, T2D before SGLT2i treatment; T2D+S12, T2D with SGLT2i treatment for 12 months.

As shown in **Figure 1A**, small RNA sequencing revealed changes in EV miRNA expression profiles following SGLT2i treatment. A total of 52 miRNAs were found to be up-regulated, while 39 miRNAs were down-regulated. These miRNAs showed significant changes (greater than 1.5-fold) in patients with T2D after 12 months of treatment with SGLT2i compared to those before treatment (**Figure 1B**). For validation experiments of miRNA expression, we selected 18 miRNAs that showed significant changes (more than 2-fold) in patients with T2D treated SGLT2i for 12 months compared to those before treatment (**Table 2**).

**Table 2:** Candidates satisfying criteria of 2-fold change and *p* < 0.05 by RNA sequencing analysis.

Mature hsa-miRNA	Fold change (T2D+S12/T2D+S0)	*P*-value
miR-376a-3p	6.35	<0.001
miR199a-3p	2.35	<0.001
miR-17-5p	2.18	<0.001
miR-27a-3p	1.95	0.004
miR-24-3p	1.75	0.001
miR-93-5p	1.70	0.001
miR-425-5p	1.70	0.008
miR-191-5p	1.53	0.004
miR-103a-3p	1.52	0.018
miR-423-5p	-1.68	0.014
let-7b-5p	-1.73	0.004
let-7d-3p	-2.64	<0.001
miR-193b-5p	-2.80	0.002
miR-6126	-2.80	0.003
let-7b-3p	-2.97	<0.001
miR-150-3p	-3.14	<0.001
miR-483-5p	-3.33	0.001
miR-483-3p	-6.35	<0.001

A value of p < 0.05 was considered statistically significant. Hsa, *Homo sapiens*; miR, microRNA.

### 3.3 Validation of selected miRNAs in patients treated with SGLT2i for 12 months

**Figure 2:**
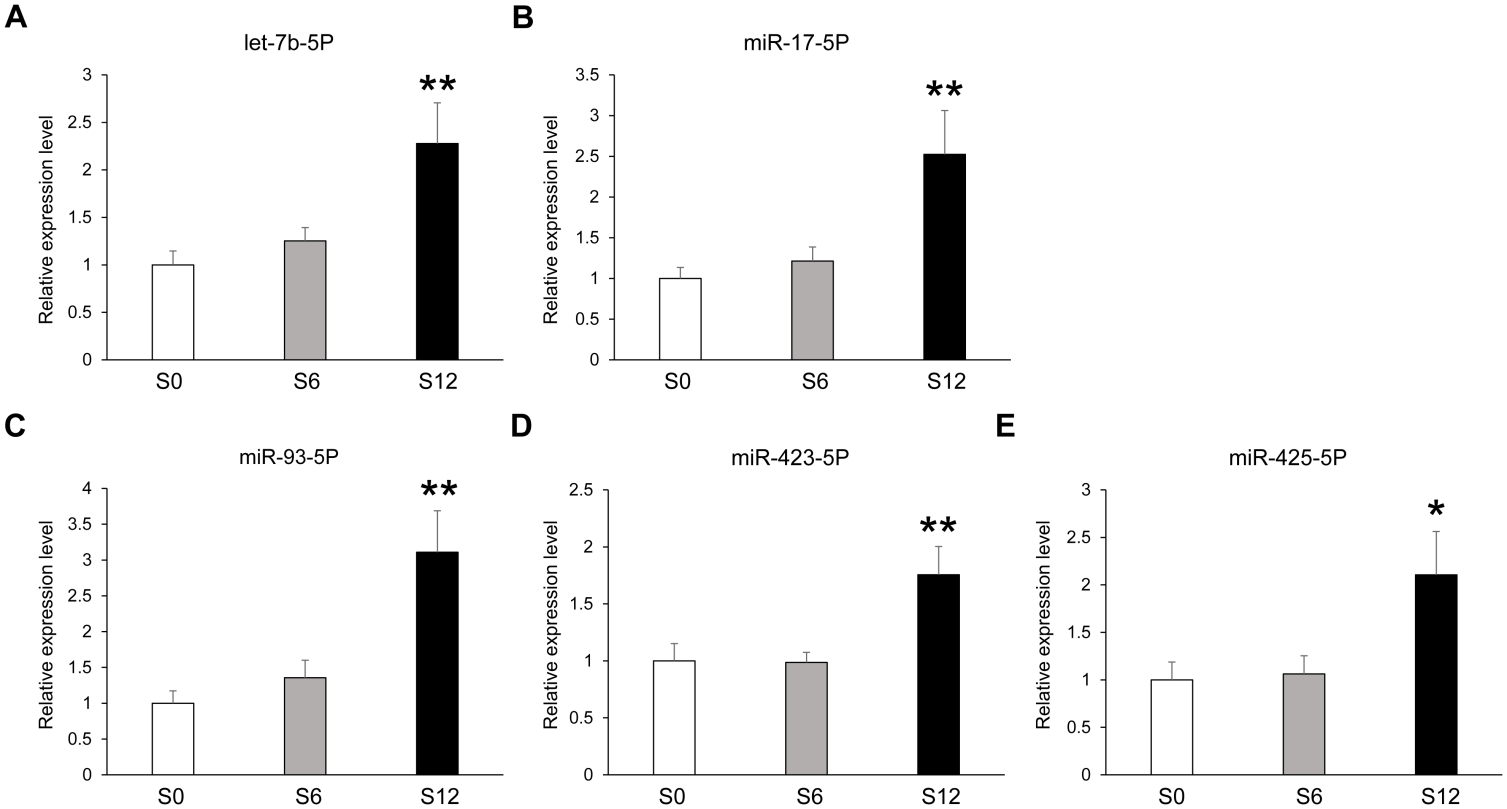
Expression levels of plasma-derived EV miRNAs in T2D patients before and after SGLT2i treatment. Expression levels of let-7b-5p **(A)** miR-17-5p **(B)** miR-93-5p **(C)** miR-423-5p **(D)** and miR-425-5p **(E)** were determined by qRT-PCR analysis. Results are shown as mean ± SEM (n = 30). *p < 0.05 and **p < 0.01 compared to baseline (S0). S0, T2D before SGLT2i treatment; S6, T2D with SGLT2i treatment for 6 months; S12, T2D with SGLT2i treatment for 12 months.

To validate the 18 candidate plasma-derived EV miRNAs identified in the screening experiment, we recruited a new cohort of 30 treatment-naive, newly diagnosed patients with T2D and analyzed EV miRNA expression at 0, 6, and 12 months after SGLT2i treatment. These patients were then co-administered with metformin and SGLT2i. For T2D patients taking SGLT2i (empagliflozin or dapagliflozin), blood samples were collected at 0, 6, and 12 months of SGLT2i treatment (**Table 3**). In the analysis of samples from three groups, treatment with SGLT2i led to notable reductions in body weight, BMI, waist circumference, fasting blood glucose, HbA1c, and systolic blood pressure. Among these, body weight, BMI, and waist circumference exhibited a progressive decline over the treatment period, while fasting glucose and HbA1c levels showed marked decreases after six months of therapy. However, they showed a tendency to recover slightly after 12 months of treatment. After 12 months of SGLT2i therapy, significant decreases were observed in fasting plasma insulin levels, the HOMA-IR index, and serum LDL cholesterol compared to baseline values. Additionally, there were substantial reductions in visceral adipose tissue (VAT), subcutaneous adipose tissue (SAT), overall fat mass, and total body fat percentage relative to pre-treatment measurements. However, total body lean mass did not show significant changes after 12 months of SGLT2i treatment compared with measurement at baseline. Validation was also performed in a separate patient cohort at 0, 6, and 12 months of therapy to evaluate time-dependent expression changes. To validate the expression of 18 selected miRNAs after SGLT2i treatment over 12 months, we measured expression levels of EV miRNAs in samples at 0, 6, and 12 months after SGLT2i treatment using qRT-PCR (**Table 4**). **Table 4** summarizes the overall ANOVA analysis results for 18 candidate EV miRNAs across S0, S6, and S12, reflecting their time-dependent expression patterns under SGLT2i treatment. Among these, five miRNAs—let-7b-5p (p = 0.0092), miR-17-5p (p = 0.0083), miR-93-5p (p = 0.0076), miR-423-5p (p = 0.0067), and miR-425-5p (p = 0.021)—were significantly upregulated after 12 months compared with baseline (**Figure 2**). While several metabolic indicators such as fasting blood glucose, HbA1c, and systolic blood pressure showed noticeable improvement by 6 months of treatment, changes in EV miRNAs were not statistically significant at that time point. Significant upregulation was only observed after 12 months, suggesting that the modulation of EV miRNA expression requires sustained exposure to SGLT2i. This indicates that the molecular response lags behind early metabolic improvements, supporting a time-dependent mechanism of action.

**Table 3:** Patient characteristics before and after SGLT2i treatment for validation experiment.

Characteristics	T2D+S0	T2D+S6	T2D+S12	*P*-value
Number (male/female)	30 (16/14)	30 (16/14)	30 (16/14)	–
Age (year)	58.95 ± 9.76	58.95 ± 9.76	58.95 ± 9.76	–
Body weight (kg)	72.67 ± 13.19	71.85 ± 13.16	70.41 ± 13.06	<0.001
BMI (kg/m^2^)	26.70 ± 3.15	26.48 ± 2.92	25.95 ± 2.81	0.001
Waist circumference (cm)	90.14 ± 8.06	88.00 ± 8.36	86.38 ± 8.16	<0.001
Fasting glucose (mg/dL)	130.81 ± 21.45	118.19 ± 11.99	124.29 ± 14.92	0.045
HbA1c (%)	7.69 ± 0.98	6.69 ± 0.37	6.92 ± 1.14	0.031
Fasting Insulin (μIU/mL)	8.67 ± 6.89	7.13 ± 6.58	6.52 ± 5.22	0.013
HOMA-IR	2.81 ± 2.06	2.26 ± 1.96	1.93 ± 1.40	0.011
BUN (mg/dL)	17.05 ± 6.79	17.08 ± 5.39	17.11 ± 5.31	0.968
Creatinine (mg/mL)	0.84 ± 0.26	0.83 ± 0.21	0.82 ± 0.25	0.321
eGFR (mL/min/1.73m^2^)	88.47 ± 24.71	90.33 ± 25.38	91.15 ± 29.27	0.357
Systolic blood pressure (mmHg)	128.10 ± 11.84	129.24 ± 13.67	122.48 ± 12.56	0.048
Diastolic blood pressure (mmHg)	73.67 ± 11.75	71.29 ± 11.19	69.81 ± 10.10	0.256
Total cholesterol (mg/dL)	167.91 ± 40.62	158.45 ± 34.28	147.66 ± 23.81	0.138
Triglyceride (mg/dL)	130.35 ± 37.90	137.21 ± 35.83	147.78 ± 40.84	0.369
HDL (mg/dL)	50.97 ± 12.81	51.22 ± 11.46	51.88 ± 13.87	0.436
LDL (mg/dL)	92.65 ± 34.79	83.16 ± 28.37	75.74 ± 22.25	0.044
AST (U/L)	23.00 ± 6.80	25.00 ± 5.94	27.00 ± 7.91	0.143
ALT (U/L)	23.81 ± 8.51	25.19 ± 9.23	28.00 ± 7.65	0.129
VAT Area (cm^2^)	157.77 ± 8.47	148.23 ± 9.17	135.89 ± 7.36	0.002
SAT Area (cm^2^)	19.60 ± 0.56	19.46 ± 0.61	19.39 ± 0.58	0.048
Total body fat mass (kg)	24.78 ± 1.20	23.84 ± 1.17	23.17 ± 1.13	0.001
Total body fat percentage (%)	37.04 ± 1.42	36.05 ± 1.13	35.90 ± 1.46	<0.001
Total body lean mass (kg)	38.95 ± 1.82	39.18 ± 1.72	39.74 ± 1.90	0.583

The validation cohort consisted of the same 30 patients followed longitudinally at baseline, 6 months, and 12 months after initiation of SGLT2i therapy. Therefore, demographic parameters such as mean age remained constant across all time points. Values are expressed as number or mean ± SD. A value of p < 0.05 was considered statistically significant. HbA1c, hemoglobin A1c; HOMA-IR, homeostatic model assessment of insulin resistance; M/C ratio, Microalbumin creatinine ratio; BUN, blood urea nitrogen; eGFR, estimated glomerular filtration ratio; VAT, visceral adipose tissue; SAT, subcutaneous adipose tissue; T2D+S0, type 2 diabetes before SGLT2i treatment; T2D+S6, T2D after 6 months of SGLT2i treatment; T2D+S12, T2D after 12 months of SGLT2i treatment.

**Table 4:** Expression of plasma-derived EV miRNAs in patients before and after SGLT2i treatment by qRT-PCR analysis.

Mature hsa-miRNA	T2D+S0	T2D+S6	T2D+S12	*P*-value
let-7b-5p	1.00 ± 0.67	1.25 ± 0.64	2.28 ± 1.97	0.009
miR-17-5p	1.00 ± 0.62	1.21 ± 0.79	2.52 ± 2.46	0.012
miR-93-5p	1.00 ± 0.79	1.35 ± 1.12	3.11 ± 2.66	0.002
miR-423-5p	1.00 ± 0.69	0.99 ± 0.41	1.76 ± 1.13	0.001
miR-425-5p	1.00 ± 0.86	1.06 ± 0.88	2.11 ± 2.09	0.016
let-7b-3p	1.00 ± 2.54	0.56 ± 1.12	0.81 ± 1.99	0.566
let-7d-3p	1.00 ± 0.80	0.88 ± 0.35	1.24 ± 1.08	0.317
miR-24-3p	1.00 ± 0.74	1.02 ± 0.52	1.66 ± 2.26	0.227
miR-27a-3p	1.00 ± 1.08	0.88 ± 0.89	1.33 ± 1.31	0.214
miR-103a-3p	1.00 ± 1.07	1.06 ± 1.76	1.56 ± 2.11	0.477
miR-150-3p	1.00 ± 1.53	0.88 ± 0.62	0.98 ± 0.66	0.803
miR-191-5p	1.00 ± 0.98	0.74 ± 0.44	1.47 ± 2.16	0.227
miR-193b-5p	1.05 ± 1.57	2.72 ± 6.47	0.87 ± 1.15	0.250
miR-199a-3p	1.00 ± 0.88	0.67 ± 0.43	1.39 ± 2.20	0.221
miR-483-5p	1.00 ± 1.88	1.44 ± 2.45	1.45 ± 2.49	0.060
miR-376a-3p	1.00 ± 0.99	0.94 ± 0.87	2.01 ± 4.51	0.282
miR-483-3p	1.00 ± 0.54	1.33 ± 1.29	1.19 ± 0.99	0.475
miR-6126	1.00 ± 0.80	1.05 ± 0.74	1.16 ± 0.80	0.575

Values are expressed as mean ± SEM (n = 30). A value of p < 0.05 was considered statistically significant. T2D+S0, type 2 diabetes before SGLT2i treatment; T2D+S6, T2D after 6 months of SGLT2i treatment; T2D+S12, T2D after 12 months of SGLT2i treatment.

### 3.4 Identification of predicted miRNA target genes and functional analysis

**Figure 3:**
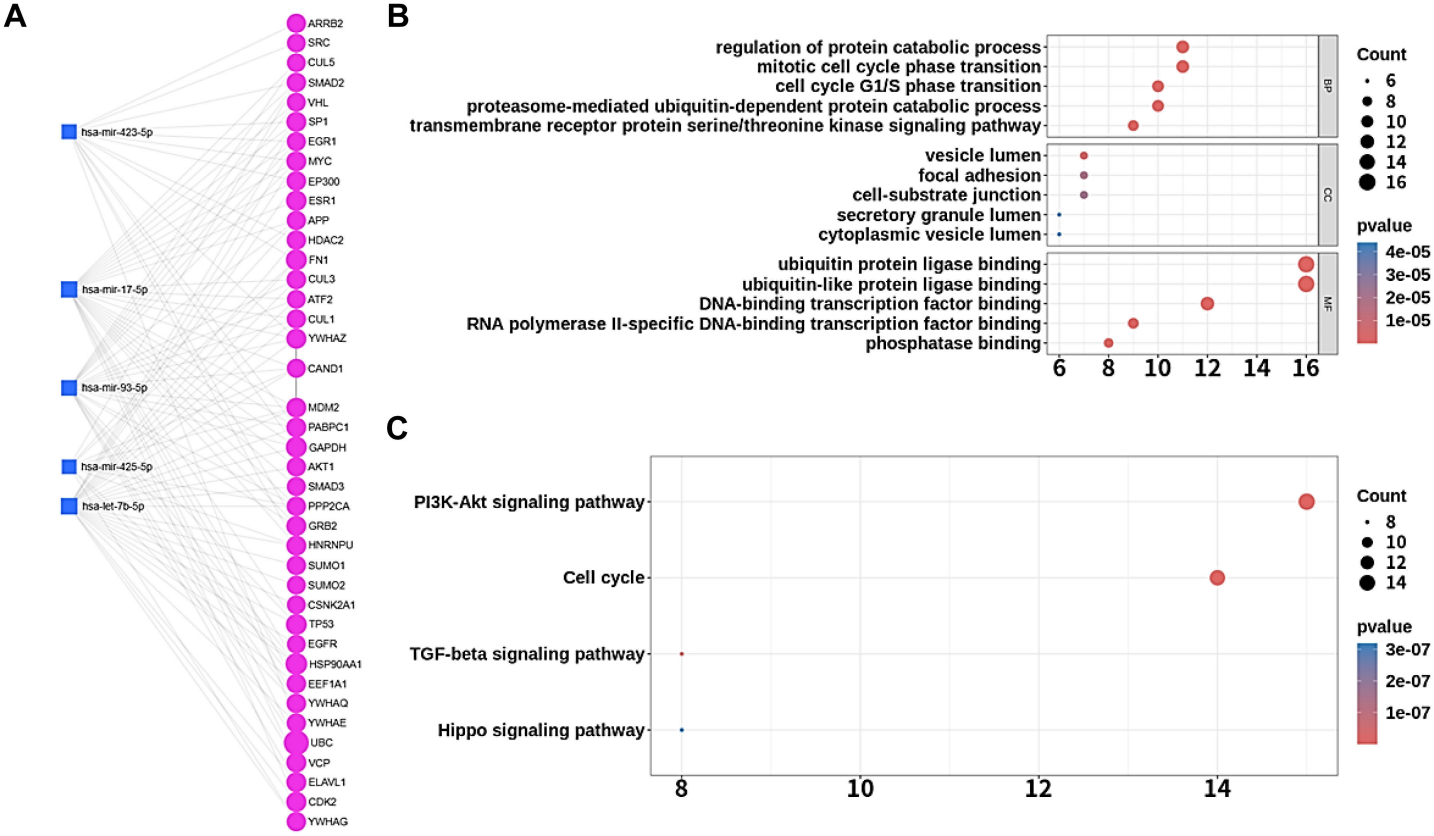
Functional and pathway enrichment analysis of miRNA-target networks. **(A)** The miRNA-target interaction network showing five key miRNAs and their associated target genes. Blue squares represent miRNAs, and magenta circles represent their target genes. **(B)** Gene ontology enrichment analysis of miRNA target genes including BP, CC, and MF. The x-axis shows gene count for each enriched term, with bubble size indicating the count and color representing the significance (*p*-value). **(C)** KEGG pathway enrichment analysis of miRNA target genes. Pathways are displayed with their significance levels (color) and associated gene counts (bubble size).

Using miRNet, we constructed an EV miRNA-target interaction network focusing on five key miRNAs identified in patients with T2D who had received a long-term SGLT2i treatment. The network comprised 40 target genes and 5 miRNAs, with 107 interactions linking these components (**Figure 3A**). Functional enrichment analysis provided insights into biological roles of miRNA-target interactions (**Figure 3B**). KEGG pathway analysis further highlighted the involvement of these miRNAs in PI3K-Akt signaling pathway, cell cycle, TGF-beta signaling pathway, and Hippo signaling pathways (**Figure 3C**). These pathways are associated with adipose tissue metabolism such as adipocyte differentiation, adipogenesis, and lipolysis.

### 3.5 Association between plasma-derived EV miRNA and fat distribution

To identify miRNAs associated with weight loss after SGLT2i treatment, we performed Spearman correlation analysis between changes in clinical parameters and expression levels of five selected miRNAs. As shown in **Table 5**, only one of the selected miRNAs, miR-425-5p, had a significant negative correlation with the reduction of VAT (p = 0.035). These results suggest that miR-425-5p may contribute to the underlying mechanism by which SGLT2 inhibitors promote weight loss.

**Table 5:** Spearman correlation between clinical parameters and selected miRNAs.

miRNAs	VAT	SAT	Total fat mass	Total fat percentage	Lean body mass
let-7b-5p					
Correlation coefficient R	-0.453	-0.196	-0.380	0.002	-0.437
*p*-value	0.104	0.503	0.180	0.994	0.118
miR-17-5p					
Correlation coefficient R	-0.532	0.007	-0.301	0.088	-0.341
*p*-value	0.053	0.982	0.296	0.764	0.233
miR-93-5p					
Correlation coefficient R	-0.376	0.029	-0.257	0.046	-0.165
*p*-value	0.185	0.923	0.375	0.875	0.573
miR-423-5p					
Correlation coefficient R	-0.535	-0.156	-0.437	-0.232	-0.200
*p*-value	0.052	0.594	0.118	0.426	0.493
miR-425-5p					
Correlation coefficient R	-0.565	-0.090	-0.116	0.223	-0.398
*p*-value	0.035	0.759	0.692	0.444	0.159

A value of p < 0.05 was considered statistically significant. VAT, visceral adipose tissue; SAT, subcutaneous adipose tissue.

### 3.6 Roles of miR-425-5p in adipogenesis and lipogenesis in 3T3-L1 cells

**Figure 4:**
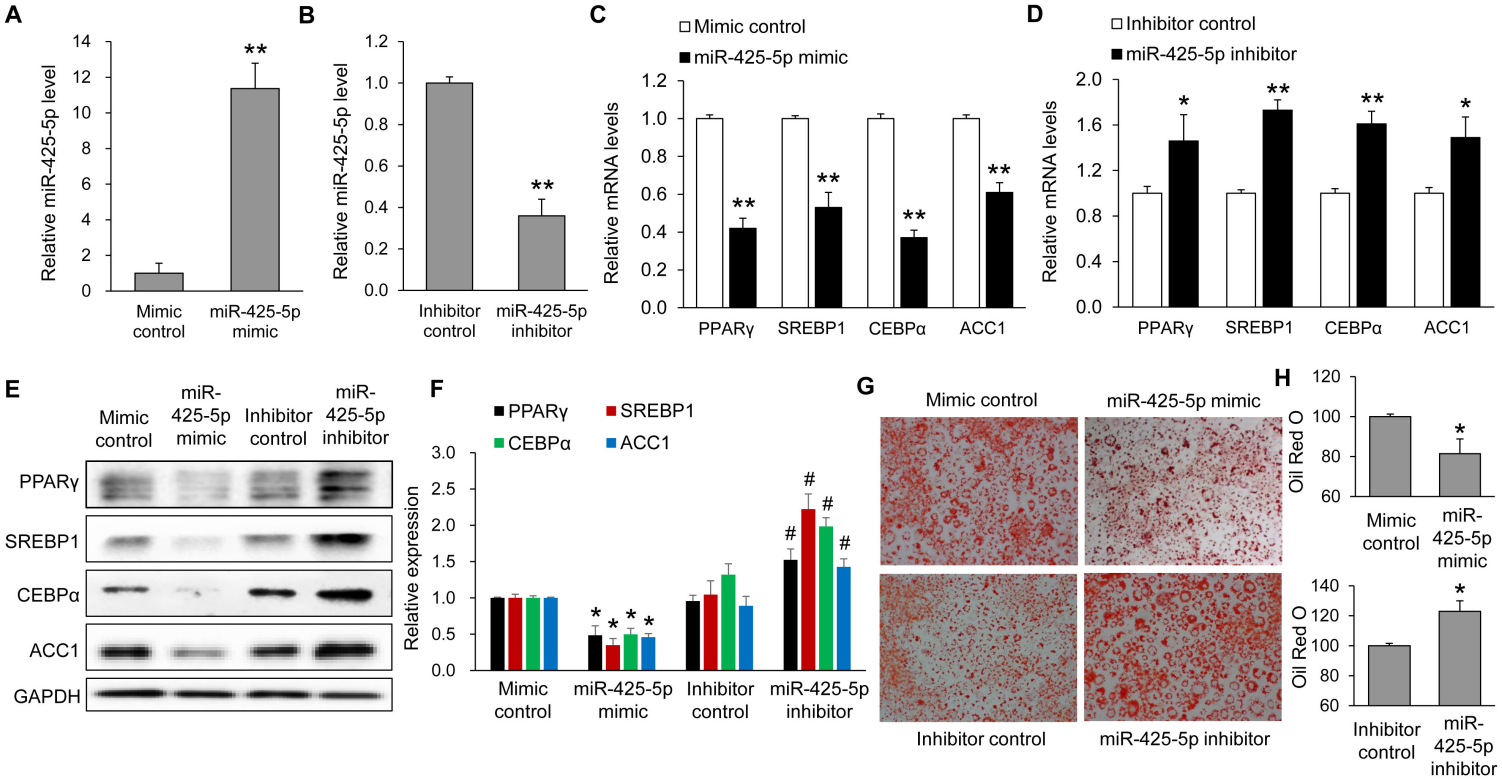
Roles of miR-425-5p in adipogenesis in 3T3-L1 cells. Expression levels of miR-425-5p were evaluated after transfection with mimic **(A)** or inhibitor **(B)**. qRT-PCR was performed to assess the relative mRNA levels of PPARγ, SREBP1, CEBPα, and ACC1 after transfection with mimic **(C)** or inhibitor **(D)** of miR-425-5p. **(E)** Representative images of Oil Red O staining taken at 8 days after induction of differentiation. **(F)** Quantitative lipid accumulation was determined by eluting the Oil Red O precipitate with isopropanol. **(G)** Western blot analysis of PPARγ, SREBP1, CEBPα and ACC1 proteins in transfected cells. **(H)** Band intensities were quantified using ImageJ software and normalized to GAPDH. All values are presented as relative levels normalized to the control (set as 1.0) and expressed as mean ± SEM, including control groups, from three independent experiments (n = 7). *p < 0.05 and **p < 0.01 *vs*. each control. #p < 0.05 *vs*. inhibitor control.

Compared with each control, transfection with the miR-425-5p mimic induced an elevation in its expression (**Figure 4A**), while transfection with the miR-425-5p inhibitor induced a reduction (**Figure 4B**). To ensure that the concentration of miR-425-5p used for functional assays did not induce cytotoxicity, we performed a CCK-8 viability assay in 3T3-L1 preadipocytes. As shown in **Supplementary Figure S2**, miR-425-5p mimic or inhibitor up to 100 nM for 72 h did not reduce cell viability compared with RNAiMAX-only controls. In addition, at 50 nM, no time-dependent changes in viability were observed between 6 and 72 h post-transfection (p > 0.05). These findings confirm that 50 nM is a non-toxic and appropriate concentration for subsequent experiments. Overexpression of miR-425-5p significantly decreased the expression of adipogenesis marker genes such as PPARγ and SREBP1 and lipogenesis genes such as CEBPα and ACC1 compared to overexpression of the mimic control (**Figures 4C, G, H**). Knockdown of miR-425-5p significantly increased the expression of those genes (**Figures 4D, G, H**). Compared with each control, miR-425-5p overexpression reduced intracellular lipid levels, whereas its inhibition increased lipid accumulation (**Figures 4E, F**).

### 3.7 Role of DACT1 as a direct target of miR-425-5p in adipocytes

**Figure 5:**
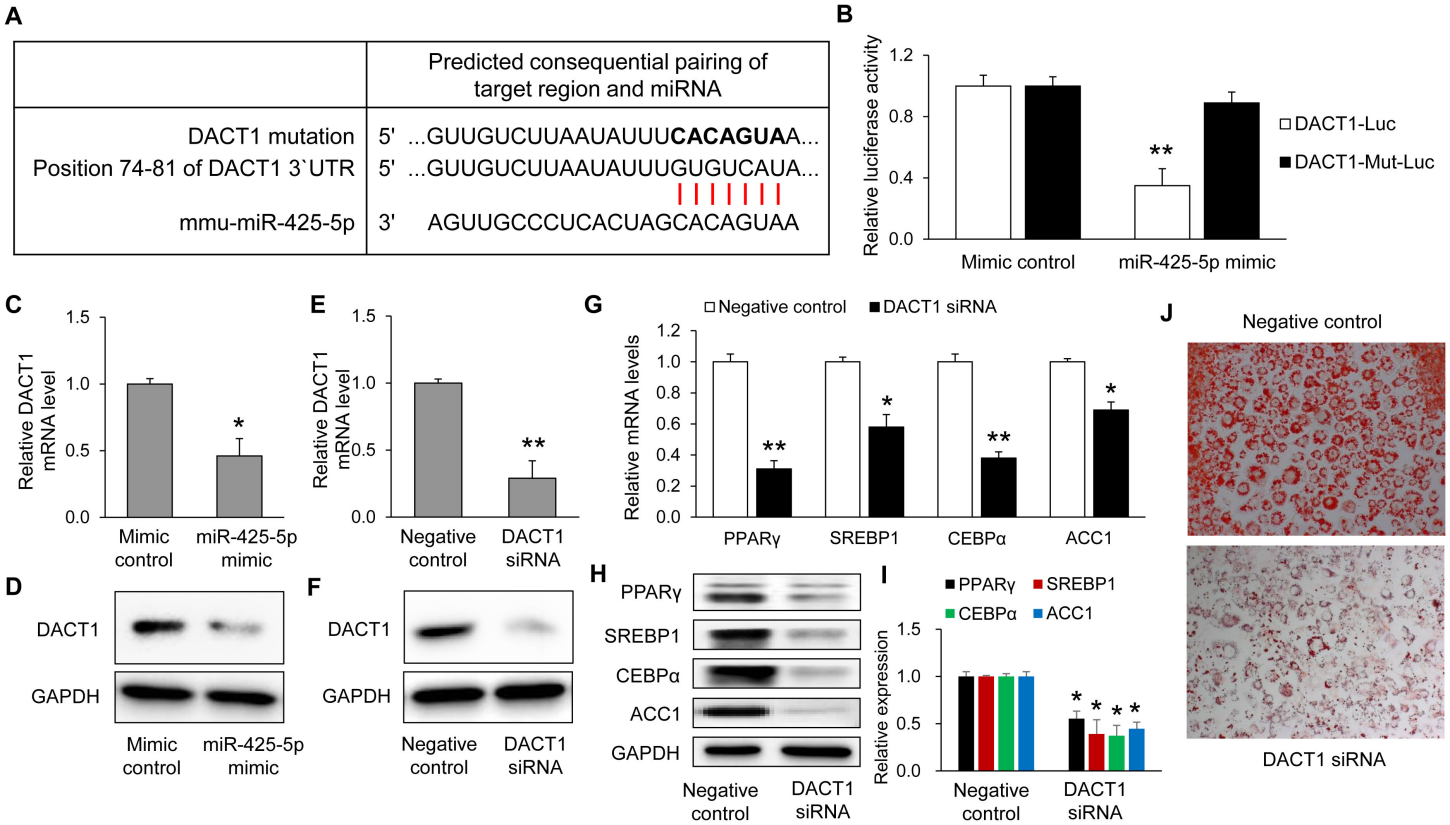
DACT1 as a direct target of miR-425-5p in 3T3-L1 adipocytes. **(A)** Schematic representation of the predicted miR-425-5p binding site in the 3′UTR of mouse DACT1 mRNA and the corresponding mutation design. **(B)** Luciferase reporter assays were performed after co-transfection of mutant DACT1 constructs and miR-425-5p mimic in HEK293 cells. After transfection with miR-425-5p mimic in 3T3-L1 cells, mRNA **(C)** and protein **(D)** expression levels of DACT1 were determined. DACT1 expression was confirmed at the mRNA **(E)** and protein **(F)** levels by transfecting 3T3-L1 cells with DACT1-specific siRNA. The expression of adipogenic markers PPARγ, SREBP1, CEBPα, and ACC1 was evaluated post-transfection at both the mRNA **(G)** and protein **(H)** levels. **(I)** Quantification of Western blot signals was performed using ImageJ, and relative protein expression levels were normalized to GAPDH. **(J)** Representative images of Oil Red O staining taken 8 days after inducing differentiation. All quantitative results are shown as relative values normalized to the control (set as 1.0) and presented as mean ± SEM (including control groups) from at least three independent experiments (n = 7). *p < 0.05 and **p < 0.01 *vs*. control. Mut, mutation.

To investigate how miR-425-5p influences adipogenesis and lipogenesis in 3T3-L1 cells, potential mRNA targets were predicted using TargetScan. This analysis identified that DACT1 may be one of the direct targets of miR-425-5p (**Figure 5A**). Luciferase activity was significantly reduced by miR-425-5p overexpression, but mutation of the binding site abolished this inhibition (**Figure 5B**) in HEK293 cells. Overexpression of miR-425-5p in 3T3-L1 cells resulted in a significant decrease in both DACT1 mRNA and protein expression (**Figures 5C, D**). To further evaluate DACT1’s role, its expression was silenced in 3T3-L1 cells using siRNA. DACT1 knockdown effectively lowered DACT1 mRNA and protein levels relative to negative control cells (**Figures 5E, F**). Consistent with these results, DACT1 inhibition significantly reduced adipogenesis and adipogenic genes (**Figures 5G, H, I**), as well as a decrease in intracellular lipid accumulation (**Figure 5J**). Collectively, these results suggest that miR-425-5p modulates adipogenesis and lipogenesis, at least partially, through direct targeting of DACT1.

## 4 Discussion

miRNAs contribute to the development of T2D by modulating insulin synthesis, release, and signaling pathways (6). Certain miRNAs are involved in regulating glucose homeostasis and insulin signaling. miR-16, 106-5p, 222-3p, 375, and 7218-5p are known to target pancreatic β-cells and affect insulin secretion (27), while miR-27a, 29a, 155, 222, and 802-5p target skeletal muscle and contribute to insulin resistance (28). In addition, miR-29a, 99b, 155, and 222 are known to target the liver and contribute to glucose metabolism disorders (12, 28). These findings imply that EV miRNAs could significantly influence the development of insulin resistance and impairments in β-cell function associated with diabetes.

miRNAs are also crucial regulators in obesity and lipid metabolism, impacting processes such as adipogenesis, lipid storage, and fatty acid oxidation. For example, miR-27a and miR-143 play essential roles in adipocyte differentiation and fat cell development, contributing to the accumulation of adipose tissues often observed in obesity (29, 30). Additionally, miR-181d predominantly found in white adipose tissues can influence lipid storage and adipose tissue expansion, which are key factors in obesity pathophysiology (31). Therefore, elucidating changes and functions of plasma-derived EV miRNAs are important for understanding the pathophysiology of T2D to develop suitable therapeutic strategies.

It has been shown that SGLT2i can reduce body weight and specifically target visceral fat, a key factor in metabolic complications associated with T2D and obesity. By promoting the excretion of glucose through urine, SGLT2i can lead to a mild but steady calorie loss, contributing to gradual weight reduction (32). While weight loss effects of SGLT2i are often mild, its ability to decrease visceral fat and its metabolic benefits make SGLT2i a valuable option for managing obesity and reducing the risk of cardiovascular complications (33). In addition to overall weight loss, SGLT2i can specifically reduce visceral adipose tissue. This is particularly important given the strong link between visceral obesity and insulin resistance (34–36). In addition, SGLT2i significantly impacts lipid metabolism by promoting lipolysis and reducing lipid storage capacity in abdominal adipose tissues (37). SGLT2i can also adjust the lipogenesis-to-lipolysis ratio, contributing to a reduction in adipose tissue and a decrease of visceral fat accumulation. SGLT2i can induce upregulation of uncoupling protein 1 (*Ucp*1) and thermogenesis-related genes through the AMP-activated protein kinase (AMPK)/sirtuin 1 (SIRT1) pathway in visceral adipose tissues (38). However, the mechanism of SGLT2i in improving abdominal obesity and lipid metabolism has not yet been clearly elucidated.

Although our study did not include healthy controls for direct comparison, plasma-derived EV miR-425-5p levels were significantly elevated after 12 months of SGLT2 inhibitor therapy compared with pretreatment values, indicating a treatment-associated molecular adaptation. This delayed upregulation suggests that long-term SGLT2 inhibition gradually modulates EV miRNA composition, reflecting sustained metabolic improvement in patients with T2D. While prior clinical data on plasma (non-EV) miR-425-5p levels in T2D remain limited, emerging reports have begun to describe altered circulating concentrations of this miRNA in diabetic conditions. Together with our longitudinal data, these observations imply that miR-425-5p could serve as a potential biomarker reflecting improved adipose-tissue metabolism and systemic insulin sensitivity following SGLT2 inhibitor therapy. Further large-scale studies are warranted to validate these associations and determine their clinical significance.

SGLT2 inhibitors are known to induce systemic metabolic remodeling beyond glucose lowering. Chronic SGLT2 inhibition promotes glucosuria and mild caloric loss, which lead to reductions in circulating insulin and leptin levels and enhancement of lipid oxidation in adipose tissue and liver. These systemic effects can alter the metabolic and redox status of multiple tissues, thereby influencing extracellular vesicle biogenesis and miRNA cargo loading. Because EV release and miRNA composition reflect the cellular state of energy balance, oxidative stress, and inflammation, the long-term metabolic adaptation triggered by SGLT2 inhibition likely contributes to the observed changes in plasma-derived EV miRNA expression. The delayed upregulation of EV miRNAs such as miR-425-5p after 12 months of therapy may therefore represent a secondary adaptive communication signal following sustained improvement in insulin sensitivity and adipose tissue function, rather than a direct pharmacologic action on SGLT2 itself. This interpretation supports the concept that EV miRNA modulation is part of the broader network of metabolic reprogramming induced by SGLT2 inhibition. This interpretation aligns with reports showing that metabolic interventions—such as caloric restriction and AMPK activation—can dynamically reprogram EV miRNA cargo composition, reflecting improved cellular energy homeostasis.

Furthermore, our data showed that several clinical parameters, including fasting glucose, HbA1c, and systolic blood pressure, showed significant or near-significant improvements after 6 months of SGLT2i treatment, whereas the expression of plasma-derived EV miRNAs, including miR-425-5p, remained unchanged for up to 12 months. This delayed increase suggests that miRNA regulation may be a secondary adaptive response to sustained metabolic improvements rather than an initial pharmacological effect. This time-dependent regulation suggests that long-term SGLT2 inhibition induces a progressive remodeling of metabolic signaling networks, which is ultimately reflected at the molecular level of EV cargo composition.

The delayed upregulation of specific EV miRNAs following 12 months of SGLT2i therapy likely reflects an adaptive molecular response to prolonged metabolic modulation. By promoting glucosuria, increasing fatty-acid oxidation, and reducing insulin levels, SGLT2 inhibition initiates adipose-tissue remodeling and changes in inter-organ communication. These systemic changes may gradually alter the release and cargo of EVs from adipose, hepatic, and renal cells, thereby modifying circulating EV miRNA profiles. Accordingly, the observed increase in plasma-derived EV miR-425-5p may represent a downstream signature of improved metabolic homeostasis rather than an immediate drug effect.

It has been reported that SGLT2i can decrease miRNA-34a-5p expression, which can induce downregulation of GREM2 to inactivate hepatic stellate cells of non-alcoholic fatty liver disease (39). In addition, decreased miR-21 in patients with diabetic cardiomyopathy can be increased by SGLT2i treatment (22). Although miRNA-34a-5p and miR-21 have been reported to have functions in lipid metabolism and obesity, their associations with weight loss and improvement of lipid metabolism by SGLT2i have not been reported yet. In this study, five extracellular vesicle-associated miRNAs including miR-425-5p showed significant alterations in the plasma of T2D patients after 12 months of SGLT2i therapy compared to baseline levels. We present novel findings of plasma-derived EV miRNAs increased by long-term SGLT2i treatment. Further studies are needed to elucidate their roles in T2D.

In this study, we identified miR-425-5p, one of five plasma-derived EV miRNAs upregulated after 12 months of SGLT2i therapy, as a strong inverse correlation with waist circumference and visceral fat levels. The mature miR-425-5p sequence is highly conserved across mammalian species (40, 41), suggesting evolutionary conservation of its metabolic functions. Although its role in obesity has not been fully elucidated, accumulating evidence links miR-425-5p to adipose-tissue biology. Consistent with our findings, Chen et al. (41) reported that miR-425-5p inhibits the differentiation and proliferation of porcine intramuscular pre-adipocytes, leading to reduced lipid accumulation. Likewise, Liu et al. (42) demonstrated that cancer-cell-derived exosomal miR-425 suppresses lipid storage in adipocytes *in vitro*. Together, these reports and our data from 3T3-L1 cells support a conserved role for miR-425-5p as a negative regulator of adipocyte differentiation and lipid storage, implying its potential relevance in obesity and adipose tissue remodeling under SGLT2i treatment.

We identified DACT1 as one of the direct targets of miR-425-5p in adipocytes. Our study provides a potential new insight that down-regulation of DACT1 by miR-425-5p may contribute to the suppression of adipocyte differentiation and lipid formation. Since miRNAs are known to regulate multiple mRNA targets, this finding suggests that miR-425-5p may influence adipocyte metabolic processes partly through targeting DACT1. Supporting this mechanism, prior research has demonstrated that DACT1 knockdown can suppress adipogenesis by activating the Wnt/β-catenin signaling pathway (43). This signaling cascade is well established as a key regulator of adipocyte differentiation and lipogenesis (44).

Our findings suggest that miR-425-5p may exert metabolically protective effects by limiting excessive lipid accumulation in adipocytes. Lipid overload in adipose tissue contributes to adipocyte hypertrophy, hypoxia, and local inflammation, which collectively drive systemic insulin resistance. In contrast, restricting abnormal adipogenesis can reduce ectopic lipid deposition and improve insulin sensitivity. Long-term SGLT2i therapy is known to promote the remodeling of dysfunctional adipose tissue, favoring smaller and more insulin-sensitive adipocytes. Therefore, the observed suppression of lipid accumulation and adipogenesis by miR-425-5p is likely beneficial rather than detrimental, as it may prevent pathological fat expansion and support metabolic homeostasis. This interpretation aligns with recent evidence showing that controlled inhibition of adipogenesis enhances insulin responsiveness in obese and diabetic models (45). Collectively, these results indicate that the miR-425-5p–DACT1 axis may contribute to the metabolic benefits of SGLT2 inhibitors through the modulation of adipose tissue quality rather than its quantity.

While our study focused on plasma-derived EVs, emerging evidence suggests that urinary EVs may provide complementary insights into diabetes-related pathophysiology. Urine represents a distinct biofluid that reflects kidney function and tubular secretion, and SGLT2i act primarily in renal proximal tubules. Therefore, profiling EV miRNAs in urine could reveal renal-specific molecular responses to SGLT2i treatment that are not captured in plasma. Indeed, the miRNA composition of urinary EVs differs substantially from that of circulating EVs (46–48), indicating tissue- and origin-specific cargo signatures. Unfortunately, urine samples were not available for all participants in the current cohort, which precluded such analysis. Nonetheless, our findings provide a foundation for future prospective studies designed to integrate plasma and urinary EV profiling to better delineate systemic versus renal contributions to SGLT2i-induced metabolic improvement.

Taken together, our findings suggest a possible mechanistic link whereby miR-425-5p, upregulated by SGLT2i treatment, could contribute to reducing visceral adiposity in T2D patients by suppressing adipocyte differentiation and lipogenesis through targeting DACT1. Therefore, long-term treatment with SGLT2i has the potential to improve abdominal obesity through miRNA regulation, suggesting that SGLT2i has broad therapeutic potential beyond glycemic control.

## Data Availability

The original contributions presented in the study are included in the article/**Supplementary Material**. Further inquiries can be directed to the corresponding author.
