# Supplementary material for: Extracellular vesicle miR-425-5p promotes visceral fat reduction via DACT1 suppression in SGLT2i-treated diabetes
Source: Front Endocrinol (Lausanne). 2025 Dec 9;16:1725625. doi: 10.3389/fendo.2025.1725625 (PMC12723516; doi:10.3389/fendo.2025.1725625)
Supplement: Supplementary file 1 [file DataSheet1.pdf]

Supplementary Material

1 Supplementary Figure

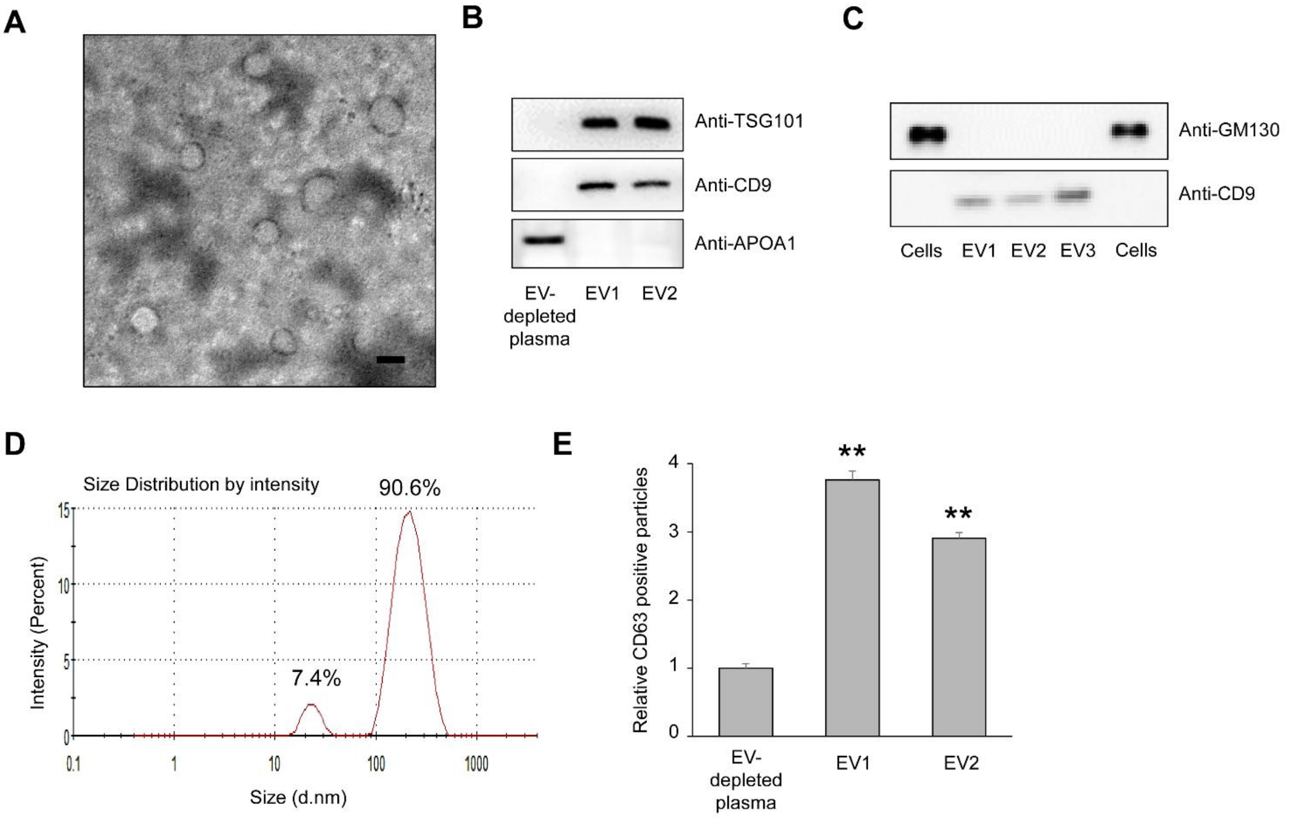

Supplementary Figure 1. Characterization of plasma-derived extracellular vesicles (EVs). (A) Transmission electron microscopy (TEM) image showing the typical round, membrane-bound morphology of purified plasma EVs. Scale bar = 200 nm. (B) Western blot analysis of EV-enriched markers (TSG101 and CD9) and the lipoprotein contamination marker APOA1 in plasma-derived EV preparations. (C) Western blot analysis demonstrating EV enrichment (CD9 positive) and absence of cellular contamination (GM130 negative) in plasma-derived EVs. (D) Dynamic Light Scattering (DLS) profile of plasma-derived EVs obtained using the Nano-ZS Zetasizer, illustrating the hydrodynamic size distribution of EV particles. (E) Quantification of CD63 expression in EV-depleted plasma and plasma-derived EV fractions using ELISA, confirming successful enrichment of CD63-positive EVs (n = 5). The values represent the mean ± SEM. $^{**}p < 0.01$ vs. control.

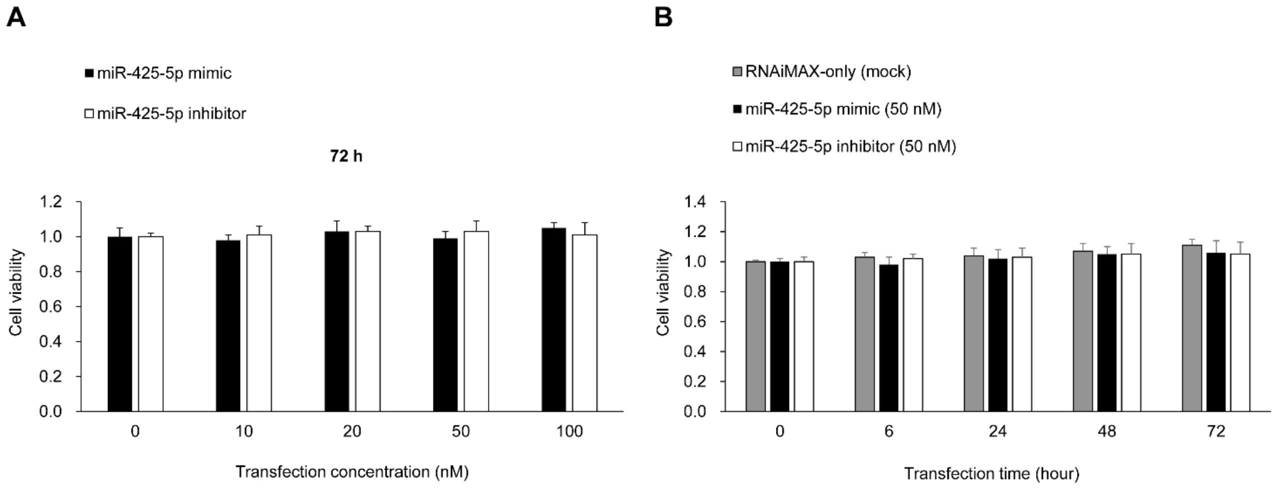

Supplementary Figure 2. Cell viability of 3T3-L1 preadipocytes following transfection with miR-425-5p mimic or inhibitor. (A) Dose-dependent analysis: Cells were transfected with 0, 10, 25, 50, or 100 nM of miR-425-5p mimic or inhibitor for 72 h, and cell viability was assessed using the CCK-8 assay. (B) Time-dependent analysis: Cells were transfected with 50 nM mimic, inhibitor, or treated with RNAiMAX only (mock control), and viability was measured at 0, 6, 24, 48, and 72 h. Data are presented as mean ± SEM (n = 6).

2 Supplementary Table

Supplementary Table S1. Summary of extracellular vesicle characterization following MISEV 2023 recommendations.

Criterion	Assessment Method	Observation / Result	Status
Morphology	Transmission Electron Microscopy (TEM)	Typical EV-like round vesicles observed	OK
Particle size distribution	Dynamic Light Scattering (DLS)	179.8 ± 73.5 nm (mean ± SD)	OK
EV surface marker (CD9, CD63, TSG101)	Western blot	Detected in EV fractions	OK
Negative marker (GM130, Golgi marker)	Western blot	Not detected in EV fractions	OK
EV protein quantification	BCA protein assay	Yield quantified for loading normalization	OK
Negative marker (ApoA1, lipoprotein marker)	Western blot	Not detected in EV fractions	OK

EV characterization was performed according to MISEV 2023 recommendations. Dynamic Light Scattering (DLS) was used for particle size measurement instead of Nanoparticle Tracking Analysis (NTA).
